# Impact of CPPs on primary human NK cell CQAs during cell expansion – an experimental study using design of experiments

**DOI:** 10.3389/fbioe.2026.1849836

**Published:** 2026-07-27

**Authors:** Gregor Mattert, Christiane Schaffer, Aleksander Szarzynski, Valentin von Werz, Oliver Spadiut, Ralf Pörtner, Werner Dammermann

**Affiliations:** 1 Center for Translational Medicine, University Hospital Brandenburg, Brandenburg Medical School Theodor Fontane, Brandenburg an der Havel, Germany; 2 Faculty of Health Sciences Brandenburg, Brandenburg an der Havel, Germany; 3 Institute of Bioprocess and Biosystems Engineering, Hamburg University of Technology, Hamburg, Germany; 4 Technische Universität Wien, Fakultät für Technische Chemie, Vienna, Austria

**Keywords:** cytotoxicity, DoE, glucose, mathematical model, NK cells

## Abstract

Human natural killer (NK) cells represent a promising leukocyte population for use in immune cell therapies. Hematologic as well as solid tumors have been shown to respond to treatment with autologous or allogeneic NK cells. Providing NK cells in sufficient quantity and quality (i.e., efficacy, safety, identity, purity, and yield), remains a challenge. A deeper insight into the critical process parameters (CPP) and critical quality attributes (CQA) of primary NK cell culture is needed for the understanding and control of NK cell manufacturing. Due to the large number of potential CPP’s, a design of experiments (DoE) approach was used for systematic planning and reduction of the number of experiments. Primary NK cells from two donors were cultivated under batch conditions and varying CPP’s. A detailed analysis of the impact of the investigated CPP’s was conducted, and the DoE based experimental setup allowed the interpretation of the influence of each selected process parameter as well as their combination on the relevant CQAs. Based on this data, a statistical model was formulated, which allows predictions of the maximal viable cell number. This approach allows for detailed insight into the CPP’s and possible optimizations in the production of cell therapy products.

## Introduction

Human immune cells have been produced using Advanced Therapy Medicinal Products (ATMP) guidelines and successfully tested in clinical studies for a number of hematological, oncological as well as autoimmune diseases, for which still only inadequate curative therapies are available, e.g., B cell acute lymphoblastic leukemia, multiple myeloma and systemic lupus erythematosus (Ali et al., 2016; Baker et al., 2023; Dagher and Posey, 2023; Grupp et al., 2013; Mackensen et al., 2022). Amongst others, natural killer (NK) cells are increasingly used in clinical studies for the treatment of patients with various malignancies (Bae et al., 2022; Lei et al., 2025; Marin et al., 2024; Tang et al., 2022). NK cells are a promising alternative to T-cells for use in cell therapy due to their innate ability to be used allogeneic to create off-the-shelf products and due to their lower risk to cause severe adverse effects like the cytokine release syndrome as well as graft versus host disease (Balkhi et al., 2025; Merino et al., 2023). Within the, mostly patient specific, manufacturing workflow for generation of the required amount of functional NK cells, clinical-grade *ex vivo* expansion is crucial (Leung, 2014; Pörtner et al., 2019; Von Werz et al., 2024). The final *in vivo* therapeutic efficacy of a cell product depends largely on the manufacturing process (Lim et al., 2015). The challenge for clinical application therefore is to reproducibly provide a sufficient number of biologically active NK-cells, in accordance with quality manufacturing guidelines (Good Manufacturing Practice, ICH, etc.) (Fang et al., 2022; Iyer et al., 2018; Koehl et al., 2016; Rezvani, 2019; Shimasaki et al., 2020). To date, immune cell products intended for clinical trials are frequently manufactured using individually controlled and variable production processes. Quality control often relies on qualitative analytical methods, with limited tracking of temporal changes and quantitative assessment of cellular proliferation and functional state throughout the manufacturing process (Abel et al., 2018; Pörtner et al., 2017).

NK cell manufacturing remains challenging, due to inter-donor variability, the intrinsic heterogeneity of the starting material, and the inherent variability of current GMP-grade expansion processes (Fang et al., 2018). Although clinically relevant cell numbers can be generated under GMP conditions, genetic inter-donor variability on, e.g., SNP level has recently been shown to impact final NK cell yields (Kusch et al., 2025). Starting material can contain differing NK cell subpopulations, i.e., CD56^bright^ CD16^−^ vs. CD56^Dim^ CD16^+^, and thus lead to variable efficacy of the final cell product (Ladd et al., 2026). GMP-grade expansion processes use differing cell culture vessels, e.g., CliniMACS® Prodigy or G-Rex®, mandating differing in-process controls (Kusch et al., 2025; Oberschmidt et al., 2019).

To improve clinical efficacy and consistency of NK-cell products, a deeper insight of critical process parameters (CPPs) and/or specific expansion techniques on critical quality attributes (CQA) for NK cell phenotype, growth and functionality is indispensable (Koehl et al., 2016; Von Werz et al., 2024; 2025). So far, optimal concentrations for medium compounds (e.g., substrates, oxygen, serum, cytokines) within the expansion process are not sufficiently characterized for process design, despite being addressed in several studies (mostly mesenchymal stem cells, T or NK cells) (Aijaz et al., 2018; Fernández et al., 2021; Koh et al., 2022; Liu et al., 2021; Temples and Sharma, 2020; Von Werz et al., 2026) (For reviews see (Abel et al., 2018; Lipsitz et al., 2016; Von Werz et al., 2024)). The following research questions related to culture conditions can be derived: (1) How does media composition and culture conditions impact cell properties and CQAs; (2) What´s the multivariate relationship between CQAs and CPPs; (3) What phenotypic changes occur within the NK cell culture over culture time.

It is therefore essential to address these questions to design well characterized expansion techniques for immune cells. Developing robust NK cell therapies requires quality-by-design (QbD), an approach that links process conditions to product quality. Most studies investigated the effect of one or two process parameters at a time, often without systematic experimental planning. This approach results in limited informational gain and fails to account for potential interdependencies between factors. To address this limitation, a simultaneous investigation of process conditions considering especially the impact on QAs is essential, e.g., by using Design of Experiment (DoE) (Kasemiire et al., 2021). Hence, this study focuses on establishing a robust model-assisted workflow for development of NK cell upstream processing, by systematically varying metabolic factors, to resolve these raised questions, by using model based experimental design techniques.

The high variability of CPPs for NK cell expansion processes such as cultivation system, pH, dissolved oxygen (pO2), nutrients, serum, IL-spiking can all influence CQAs like VCC, yield, function and phenotype of NK cells (Von Werz et al., 2024). In comparison to T-cell research, which has been fueled by the success of CAR-T cell treatments, QbD-oriented studies have been performed, that not only elucidated these relationships, but also improved existing workflows (Hood et al., 2024). By analogy, NK cell process development would also benefit from DoE based techniques for process optimization.

Among the metabolites, many exert a direct and indirect impact on NK cells. Glucose is the key nutrient in immune cell metabolism (Gardiner, 2019). Due to glycolysis lactate forms and accumulates over time in a batch system, which can inhibit NK cell activity and mimics the conditions in the tumor microenvironment (TME) (Terrén et al., 2019). Glutamine is another common metabolite in immune cell metabolism as it can be metabolized in the TCA cycle. Pyruvate is an intermediate product and can both be utilized in glycolysis or feed into the TCA cycle (Gardiner, 2019). Asparagine has been shown to influence CAR T-cell activation of effector properties and influence glucose as well as glutamine consumption (Frlic and Pavlin, 2025). Serine can be converted to formate, which plays a crucial role in one-carbon metabolism. A supplementation of one-carbon units together with formate has been shown to improve functions of exhausted CD8^+^ T-cells and induce changes in effector function and metabolic pathways (Frlic and Pavlin, 2025).

In this respect, a thorough analysis of NK cell batches with respect to their phenotype, IFN-γ production, and cytotoxicity *in vitro* is mandatory. Changes in the expression of NK cell markers can be of vital importance regarding the functionality of the cell product. This includes activating receptors, e.g., CD337 (NKp30), CD336 (NKp44), CD335 (NKp46), CD314 (NKG2D), CD226 (DNAM1) and CD16 (FCγRIII) as well as the death ligands CD253 (TRAIL) and CD178 (FasL) and exhaustion markers like CD366 (TIM-3), CD279 (PD-1), CD223 (LAG-3) and TIGIT (Bröker et al., 2019; Vivier et al., 2024; Wolf et al., 2023).

This study focuses on the basic science of NK cell upstream processing, by systematically varying metabolic factors, to resolve the raised questions, by using model based experimental design techniques. Additionally, a broad spectrum of cell surface markers was tracked throughout the experiment, linking media composition to phenotypic variation. The focus of the study was less on comparing the cells from different donors and more on establishing a robust workflow, which was tested on two donors.

## Materials and methods

### General cell culture

Purified and pre-activated NK cells from two different whole blood donors were acquired from Zellwerk GmbH as frozen stocks, 1x10^8^, each (Oberkrämer, Germany) (Bröker et al., 2019). Donor one was a 48-year-old woman with breast cancer; donor two was a healthy 26-year-old male. The NK cells were cultivated in Minimum Essential Medium Eagle - Alpha Modification (Alpha MEM) + 10% human AB serum and supplemented with 500 IU/mL IL-2 (Life Technologies, Darmstadt, Germany). IL-2 was supplemented every other day. K562-GFP cells (American Type Culture Collection, ATCC, CCL-243-GFP, Manassas, United States) and K562 (ACC-10, DSMZ German Collection of Microorganisms and Cell Cultures, Braunschweig, Germany) were cultivated in RPMI-1640 supplemented with 10% FBS. All cell culture media, human AB serum (HS) and FBS were purchased from PAN Biotech, Aidenbach, Germany.

### Batch cell culture

The cells were thawed on the same day a new experiment was started and cultivated in batch mode for 12–24 days without medium exchange. Corresponding to the experimental setting, different metabolites were spiked (see “Design of Experiments” below). Glucose, serine, and asparagine (Sigma Aldrich) were prepared as 10x stock solutions in alpha MEM according to the experimental plan (see Supplementary Table S3). Pyruvate (GIBCO) and glutamine (Sigma Aldrich) were added from stock solutions of 100 mM and 200 mM, respectively. All supplement concentrations were calculated for alpha MEM, including 10% human serum. Available cell concentrations varied due to the different cell numbers of recovered cells from frozen stocks. After thawing, NK cells were counted, divided according to the experimental plan. NK cells were washed with alpha MEM containing 10% human serum, centrifuged and resuspended in the media prepared in advance. Prior to seeding, cell concentrations were determined experimentally with cell concentrations ranging from 3 to 4.5 x10^5^ viable cells/mL. NK cells were cultivated in 24 well plates (Greiner Bio-One GmbH, Frickenhausen, Germany), 1.4 mL each well, and incubated at 37 °C with 5% CO_2_. Separate plates for each time point were prepared containing cultures with each medium condition. Experimental runs were randomized according to the run order specified by the experimental plan. Center points and medium controls were blended into the run order in a way that different positions at the center or the margins of the well plates were occupied by these controls. Empty wells were filled with the same amount of buffer. Oxygen and pH were measured continuously in separate plates using Oxodish (OD24) and Hydrodish (HD24) on a Sensor Dish Reader (PreSens Precision Sensing, Regensburg, Germany). The Sensor dishes were terminated on the last day of culture. Every second day cells were harvested for determination of viable cell count (VCC) and viability, cytotoxicity measurements, as well as for phenotypic characterization. Cell suspensions were weighed after harvesting to determine evaporation rates related to time course and well positions on the plates.

### Cytotoxicity measurements

Harvested NK cells were counted, adjusted to 2x10^6^/ml in alpha MEM with 10% human serum, and co-cultivated with K562-GFP cells in RPMI/10% FBS at a ratio of 2:1, 100 µL each. Co-cultures incubated for 4 hours at 37 °C and 5% CO_2_ (DoE1). Alternatively, K562 cells were stained with 1 µM CellTrace Violet (DoE2) according to the manufacturer’s instructions (Thermo Fisher).

Afterwards, cells were stained with PI (Sigma Aldrich Chemie, Taufkirchen, Germany) and measured on a CytoFlex flow cytometer (Beckman Coulter, Krefeld, Germany). K562 and NK cells were distinguished using the GFP or the CellTrace Violet signal of K562, respectively. Dead cells within the target cell population were identified from FSC/SSC plots according to Joslin et al. (Joslin et al., 2018). Control cultures of K562 cells only were cultured in parallel. Cytotoxicity was calculated according to the following equation: specific lysis % = (dead targets co culture % - dead targets control %)x100/(100% – dead targets control %).

### Metabolite and amino acid analyses

Cell culture supernatants and medium only controls were frozen at −80 °C and shipped to Lifespin GmbH (Regensburg, Germany). More than 30 metabolites and amino acid concentrations were determined through an NMR-based methodology. In brief, medium samples were thawed for 3 h at room temperature and mixed with equal volumes of 0.1 mol/L phosphate buffer pH 7.15 containing 5% D_2_O, 0.01% sodium azide and 6 mM pyrazine serving as an internal standard for quantification. ^1^H-NMR measurements were carried out in 5 mm Bruker NMR tubes on a Bruker AVANCE NEO 600 MHz (Bruker Corporation, Billerica, United States), using the measurement method 1D 1H noesygppr1d_d20, NS = 16, T = 310 K, for 6.5 min, each sample. A Fourier transformation was done using the TopSpin software (version 4.0, Bruker Biospin) and phase as well as baseline adjustments were carried out automatically. Metabolite concentrations were determined using the proprietary Profiler Software (Lifespin, V2.0.1_MedizinproduktBlut and V1.4_Blut, respectively). A full list of determined metabolites can be seen in Supplementary Table S3.

### ELISA measurements

Cell culture supernatants were aliquoted and stored at −80 °C. ELISA kits were used to determine concentrations of IFN-γ (#430116, Biolegend, San Diego, United States), TNF-α (#430204, Biolegend, San Diego, United States) and Granzyme B (#DY2906-05, together with #DY999, both R&D Systems, Minneapolis, United States). Sample preparation and measurements were done as recommended by the manufacturer with slight alterations. Instead of using the supplied horseradish peroxidase included in the kits, the Streptavidin-PolyHRP80 (#SP80C, SDT reagents, Baesweiler, Germany) was used. The substrate was incubated until a visible optical change occurred. Assay Diluent (#421203, Biolegend, San Diego, United States) was used for sample preparation for all ELISA Kits.

### Sample preparation for phenotypic analyses using flow cytometry

Directly after harvesting, the cells were separated for two antibody panels (Supplementary Table S3). For each experimental condition and time point, NK cells were co-cultured with K562 cells in 96 well plates for 4 h at a ratio of 2:1. Single cultures of NK cells were performed in parallel under the same conditions as controls without target cells. NK cells were adjusted to 2x10^6^/ml in alpha MEM with 10% human serum and co-cultivated with K562 cells in RPMI/10% FBS at a volume of 150 µL altogether.

The samples from single and co-culture which were to be stained with panel NK9 were incubated with GolgiPlug™ and GolgiStop™ (Beckton Dickinson, Franklin Lakes, United States). In case of co-culture samples, GolgiPlug™ and GolgiStop™ were added after the first hour of the 4-h co-culture incubation. The antibody CD107a-BV510 (Becton Dickinson, Franklin Lakes, United States) was added simultaneously with GolgiPlug and GolgiStop and cultures were kept protected from light. To allow intracellular staining, the cells were fixed and permeabilized by adding BD Cytofix/Cytoperm™ (Beckton Dickinson, Franklin Lakes, United States) and incubated for 20 min at 4 °C.

The samples from single and co-culture which were to be stained with panel NK8 were fixed using BD Cytofix™ Fixation Buffer (Beckton Dickinson, Franklin Lakes, United States) for 20 min at 4 °C following the manufacturer’s instructions.

Samples for both antibody panels were either suspended in PBS + 2% FBS at +4 °C or frozen at −80 °C in 90% FBS and 10% DMSO (Carl Roth, Karlsruhe, Germany) and shipped for flow cytometric measurements.

### Flow cytometric analyses

NK cells were suspended in a buffer containing PBS substituted with 1% (v/v) FBS (Thermo Fisher, Waltham, United States) and 0.09% (w/v) NaN3 (Merck, Darmstadt, Germany) and incubated with Human BD Fc Block™ (Becton Dickinson, Franklin Lakes, United States) for 10 min at room temperature (15 °C–25 °C) protected from light. The cells were incubated with antibodies for 30 min at room temperature (15 °C–25 °C) protected from light. Antibody volumes were determined through titration, and gate cutoffs were defined using fluorescence minus one (FMO) controls. Flow cytometric measurements were performed on a Symphony A3™ (Beckton Dickinson, Franklin Lakes, United States of America). Flow cytometric data was pre-processed using the FlowAI Plugin for FlowJo and analysed using FlowJo (v.10.10.0, Beckton Dickinson, Franklin Lakes, United States) (Monaco et al., 2016).

### Design of experiments

The experimental plan was designed according to DoE principles using MODDE® 13.1 (Sartorius, Göttingen, Germany). The first DoE (DoE-1) followed a five-factor 2-level factorial design varying glucose, glutamine, pyruvate, asparagine, and serine, i.e., glucose (low: 5 mM; middle (centerpoint): 8.75 mM; high: 12.5 mM), glutamine (low: 2 mM; middle (centerpoint): 5 mM; high: 8 mM), serine (low: 0.25 mM; middle (centerpoint): 1.13 mM; high: 2 mM), pyruvate (low: 1 mM; middle (centerpoint): 1.5 mM; high: 2 mM) and asparagine (low: 0.33 mM; middle (centerpoint): 1.17 mM; high: 2 mM). All model coefficients are displayed within a confidence interval of 95%. Additionally, uncontrolled factors pH and DO were determined online.

This resulted in a total of 41 experimental runs, including a total of nine center points at each harvest time point. A total of seven harvests for each condition were performed over 12 days. The physical execution of the experimental setup was divided into three experimental blocks, each containing three center points, and was performed using cells from donor one. Experimental runs were randomized according to the MODDE-generated run order. The center points within each block were used for data processing (see next chapter) and analyzed using MODDE® software (Supplementary Table S3).

The second DoE (DoE-2) was planned as a 3-level full-factorial design with two factors (glucose and glutamine), each at three levels, i.e., glucose (low: 12.5 mM; middle: 21.25 mM; high: 30 mM) and glutamine (low: 4 mM; middle: 8 mM; high: 12 mM). This design resulted in a total of eleven experimental runs, including three center points (Supplementary Table S3). A total of twelve harvests for each condition were performed over 24 days. DoE-2 was carried out using cells from donor two. The setup was again extended by the uncontrolled factors pH and DO.

For all DoE experiment responses, preprocessed data (see next chapter) was extracted from continuous representations at the discrete timepoint of maximum VCC. Further process relevant timepoints including: (i) maximum specific growth rate (µ_max_) and (ii) relative change within the exponential growth phase where also investigated. Furthermore, analytics on coefficient of determination, cross validation, coefficient values and their respective p-values are presented in the appendix.

### Data processing

Missing datapoints were first imputed using Kalman imputation to maintain complete time-series data trajectories. These values were primarily used for complete data trajectory representation. Furthermore, the imputed values were tracked and used during analysis as a loss weight of zero mask for model fitting, in order to only use actual observed data to produce the neural network surrogate.

A neural ordinary differential equation (NODE) approach was used to generate continuous trajectories of the time series data generated within the DoEs (Chen et al., 2019). The models were trained by including factors, responses, and an additionally measured metabolite panel (data not shown) for each DoE. A fully connected four layer, 32 neurons per layer was selected. As a loss function, β-negative log likelihood, extended with normalization of all variables and incorporating heteroscedastic uncertainty estimates, derived from the triplicate center point measurements, was used (Seitzer et al., 2022).

Originally imputed values were retained in the data matrix but were excluded from parameters fitting by zero weighting. For DoE-1, each experimental block with its unique center points was used with the corresponding data.

The center point data from these blocks collapsed by calculating the mean value of each block, which was later used for DoE-1 analysis. This was used to retain the experimental variability of each block during uncertainty estimation and to support NODE fitting. This approach was not used in DoE-2 as there was only one block with a total of three center points.

One NODE was trained on each dataset separately. Model performance was evaluated only on observed, non-masked datapoints. Originally imputed values and NODE interpolated values were excluded from the calculation of model performance metrics. Model performance and model fit metrics of the DoE-1 and DoE-2 models are reported in (appendix D).

The trained model was used to generate continuous interpolated trajectories between observed harvest points. These trajectories were then used to calculate volumetric and specific rates in a continuous manner. The resulting trajectories were used to generate one summary value per response to the DoE analytics.

Maximum values for VCC and µ were extracted directly from the NODE derived trajectories. Relative change within the exponential growth phase were extracted by selecting the exponential phase of the process, before calculating the difference in change for absolute values and average change for volumetric and specific rates (Cheng and Thrash, 2021).

### Analysis setup

All analyses were performed in a Visual Studio Code using Python 3.12.8. The NODE model was implemented in PyTorch 2.5.1, with differential equation solving performed using torchdiffeq 0.2.5 and the adjoint sensitivity method for gradient computation. Feature normalization was performed using scikit-learn’s StandardScaler 1.5.2. ODE integration during inference used SciPy’s LSODA solver via the torchdiffeq scipy_solver backend. Adam optimizer was used for training the model (1,000 iterations). Data processing was handled with Pandas 2.2.3 and NumPy 1.26.4. All computations were executed on a Windows-based workstation equipped with an Intel® Core™ i7-14700K CPU, NVIDIA GeForce RTX 4060 Ti GPU, and 32 GB RAM.

### Data visualization and statistics

Statistical analyses were performed using GraphPad Prism (v.9.5.1, GraphPad Software, Boston, United States). Graphs were created using GraphPad Prism and RStudio (v.2024.04.2, Posit Software, Boston, United States) with R (v.4.4.1, R Core Team, Vienna, Austria).

## Results

### Overview of the first DoE (DoE-1)

The pre-activated NK cells of donor one were cultivated for 12 days in batch mode. Based on consumption rates determined in a preliminary experiment (data not shown), concentrations for all varied parameters were chosen such that the low concentration would be depleted halfway through cultivation, while the middle concentration would be exhausted by its end. The high concentration was set above the expected demand to reduce the risk of nutrient limitation during cultivation. Glutamine, serine, pyruvate, and asparagine were chosen as variable parameters because preliminary experiments with the NK92 cell line indicated that these metabolites were consumed during cultivation (data not shown). Experiments with this cell line were used to define a biologically plausible starting design space for the primary NK cell DoE-1.

**FIGURE 1:**
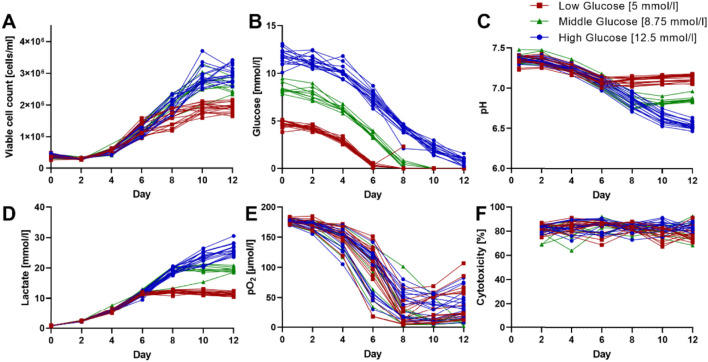
Experimental design of DoE-1. Time course of viable cell count **(A)**, glucose concentration **(B)**, pH **(C)** lactate concentration **(D)**, pO2 **(E)** and cytotoxicity against K562 cells **(F)** of NK cells of donor one, cultivated for 12 days as a batch. Experimental runs are colored according to the initial glucose concentration level. All 41 experimental setups from DoE-1 are shown for the whole cultivation period.

Glucose concentration had a clearly visible impact on cell growth with lower numbers of the VCC for the lower glucose level (Figure 1A). Final cell concentrations ranged between 1.6 and 2x10^6^ cells/mL in these experiments. Glucose was fully consumed between day 6 and 8 at these low levels (Figure 1B), which had a direct impact on lactate production and the changes in pH level (Figures 1C,D). Notably, NK cells cultivated at low glucose only stopped growing on day 10 despite glucose being completely consumed.

NK cells cultivated with high glucose levels did not run into any limitation, but cellular growth slowed towards the end of the cultivation period with increasingly higher lactate concentrations and lower pH values. Final cell concentrations ranged from 2.6 to 3.3x10^6^ cells/mL on day 12.

The cytotoxicity remained stable between 75% and 90% over the cultivation process, even though some cell cultures were approaching the stationary growth phase and had either no glucose left or dropped to a pH value below 6.5 (Figure 1F). Oxygen concentrations were strongly reduced, leading to hypoxic conditions in some culture settings (Figure 1E). This appears to be independent of the glucose level. The other varied parameters had no comparable impact on the observed parameters or quality attributes in the tested concentration ranges as glucose.

## Overview of the second DoE (DoE-2)

**FIGURE 2:**
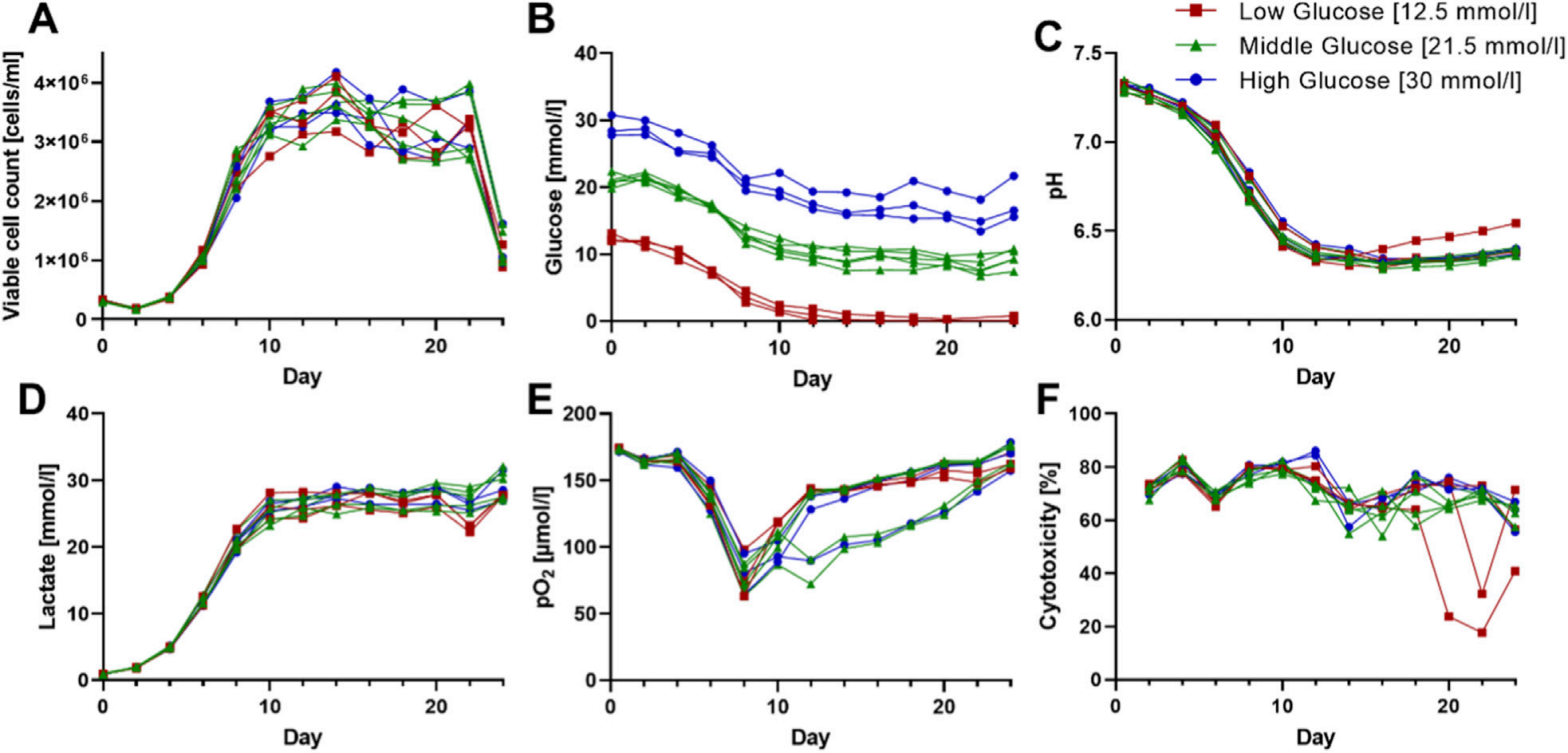
Experimental design of DoE-2. Time course of viable cell count **(A)**, glucose concentration **(B)**, pH **(C)** lactate concentration **(D)**, pO2 **(E)** and cytotoxicity against K562 cells **(F)** of NK cells of donor two, cultivated for 24 days as a batch. Experimental runs are colored according to the initial glucose concentration level. All eleven experimental setups from DoE-2 are shown over the full cultivation period.

In a second DoE (DoE-2) pre-activated NK cells from donor two were used in a similar setup with specific modifications. This time, only glucose (low: 12.5 mM; middle: 21.5 mM; high: 30 mM) and glutamine (low: 4 mM; middle: 8 mM; high: 12 mM) concentrations were varied and with overall higher concentrations to avoid an earlier limitation. The cultivation period was doubled to get a more pronounced exponential and stationary growth phase of the NK cells. Even though glucose was depleted at the lowest initial concentration level, it did not appear to be a limiting factor with respect to cell growth (Figures 2A,B). Cell growth stopped after 12–14 days, and higher cell concentrations were reached compared to DoE-1, with 2 to 3x10^6^ cells/mL in DoE-1 and 3.6 to 3.8x10^6^ cells/mL in DoE-2. Lactate concentrations and pH levels were more similar in all experimental runs compared to DoE-1 (Figures 2C,D), reaching up to 30 mM lactate and pH values below 6.4. Interestingly, despite higher cell numbers, oxygen concentrations did not deplete as much as in DoE-1 in the exponential growth phase (Figure 2E). The cytotoxicity remained stable, between 65% – 80%, even during the transition into the stationary growth phase and only dropped after day 12 (Figure 2F).

## Comparisons between both DoEs

**FIGURE 3:**
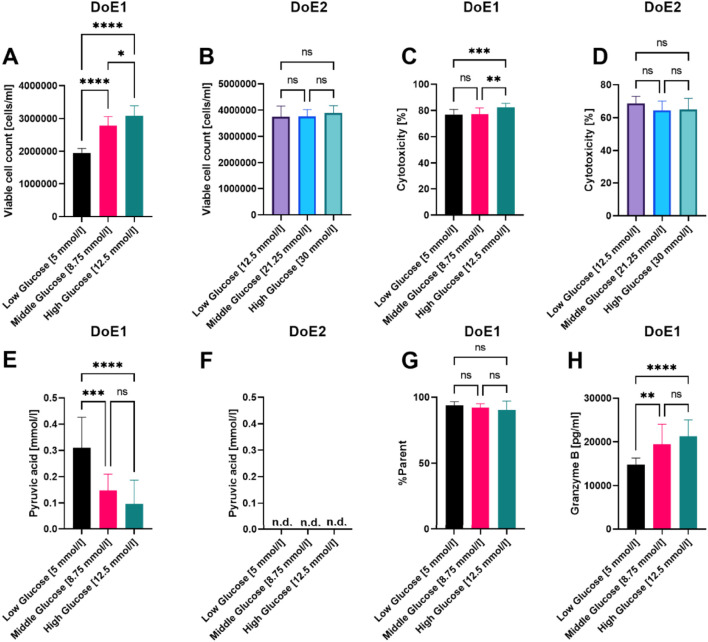
Overview of glucose-dependent differences of quality attributes at the time point of the highest viable cell count. **(A)** + **(B)** Viable cell counts by glucose level of donor one and donor two, respectively. **(C)** + **(D)** Cytotoxicity of NK cells against K562 cells by glucose level of donor one and donor two, respectively. **(E)** + **(F)** Pyruvate by glucose level of donor one and donor two. The pyruvate concentrations were below the lower limit of detection for donor two. **(G)** Percent of CD56 expressing Lymphocytes at the time point VCCmax. **(H)** Extracellular granzyme **(B)** concentration of the single culture by glucose concentration. Significances were determined using an ordinary one-way ANOVA with multiple comparisons. p > 0.05 is visualized as ns, p < 0.05 as *, p < 0.01 as **, p < 0.001 as *** and p < 0.0001 as ****. Error bars represent the standard deviation of all experimental runs separated by initial glucose level (low, middle, high) and DoE.

For DoE-1, at the time point with the highest viable cell count (VCC_max_) there were significant differences in the average VCC depending on the initial glucose concentration (Figure 3A) with a mean of 2x10^6^ cells/mL in cultures with low glucose and a mean of 3.1x10^6^ cells/mL in cultures with high glucose. This is probably linked to growth limitations for the lower and middle glucose level, since in DoE-2, where glucose was not a limiting factor, there were no significant differences (Figure 3B). Longer cultivation in DoE-2 led to higher cell concentrations. A minor influence of the initial glucose concentration on cytotoxicity cannot be excluded, given that significant differences emerged in DoE-1, while they were less distinct in DoE-2 (Figures 3C,D). Pyruvate uptake appears to be dependent on extracellular glucose concentrations and is higher in culture conditions with abundant glucose (Figure 3E). The pyruvate concentration at the time point of VCC_max_ appears to be more dependent on the initial glucose concentration than on the initial pyruvate concentration. The pyruvate concentration was below the lower limit of detection at VCC_max_ for the second set of experiments, which further indicates this dependency since glucose levels were higher in the second set. (Figure 3F). Higher glucose concentration led to a higher secretion of the cytotoxic molecule granzyme B during the single culture (Figure 3H).

## Modeling of the viable cell count

**FIGURE 4:**
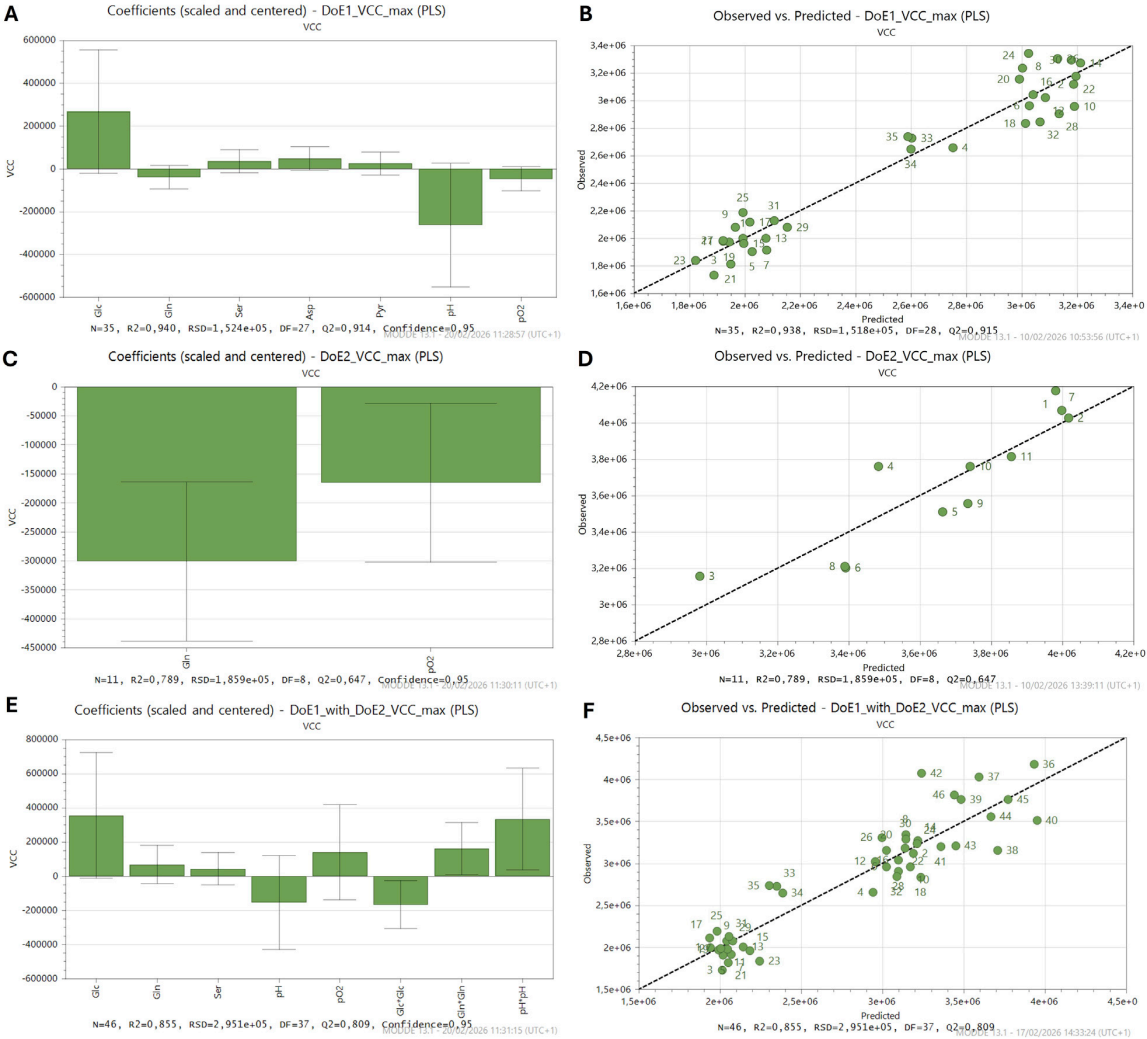
MODDE® based analysis of DoE-1 **(A,B)** with the factors glucose (Glc), glutamine (Gln), serine (Ser), pH and dissolved oxygen (pO2), DoE-2 **(C,D)** and DoE-1 extended by DoE-2 **(E,F)** on the response of maximal viable cell count (VCC_max_) using partial least squares models. On the left-hand side **(A,C,E)**, the coefficients are shown for each model. A minimal threshold of 0,6 was used to exclude coefficients to increase Q^2^. Significant and insignificant parameters are shown for each model. On the right-hand **(B,D,F)** side predicted versus observed plots are shown for VCC_max_.

Partial least squares (PLS) models were created built in MODDE® to analyze both DoE experiments. Model performance and variable selection are reported below, while full model diagnostics, coefficient tables and contour plots are provided in appendix A-B. For DoE-1, a PLS model was able to achieve a R^2^ = 0.938 and Q^2^ = 0.915 for VCC_max_, after removing pyruvate as a factor (Figure 4B). The remaining coefficients were close to statistical significance (p < 0.05) and were therefore retained as factors (Figure 4A). By excluding all factors but glucose, the model maintained high significance and explanatory power (R^2^ = 0.797, Q^2^ = 0.779; see appendix A 1.1). Because not all cultures within DoE-1 were able to reach the stationary phase within the planned 12-day period, glucose availability dominated as a VCC growth influencing factor in DoE-1.

Based on observed metabolite trends reported above and prior literature (Terrén et al., 2019; Yoo et al., 2020), glutamine was retained as a factor for the follow-up experiment DoE-2, despite borderline significance. Analyzing the PLS model for DoE-1 retained pO2 and pH as significant factors, associating them with differences in VCC at µ_max_ (appendix A 1.2). In contrast, glutamine and asparagine emerged as important metabolic factors when modelling the relative change in VCC during the exponential growth phase (appendix A 1.3).

In DoE-2 glucose and glutamine were varied at higher concentrations and cells were cultured for a longer time period, to ensure that all cultures reached stationary phase. The model for VCC_max_ showed that glutamine was a more important predictor than glucose, with R^2^ 0.789 and Q^2^ of 0.647, after removal of the non-significant factors (Figures 4C,D). Considering the results from DoE-1, it can be implied that a glucose-based limitation had been reached, and VCC was more influenced by glutamine over the experiment. A plausible explanation is the accumulation of lactate from glycolysis, which can inhibit cell growth after reaching a threshold (Von Werz et al., 2026). Modelling VCC at µ_max_ showed a similar result, where only glutamine emerged as the dominant metabolic factor (appendix B 2.2). No model was fit for the relative change within the exponential growth phase on VCC (appendix B 2.3).

In the final step, data from DoE-1 and DoE-2 were combined and modeled together to assess response patterns across both datasets. Glucose showed the strongest association with VCC_max_ (R^2^ = 0.855, Q^2^ = 0.809, Figures 4E,F), indicating a consistent glucose-based trend within the combined datasets. For the combined dataset, no model could be fit to VCC at µ_max_ or relative change in the exponential growth phase.

### Phenotypic characterization of the NK cells during culture

**FIGURE 5:**
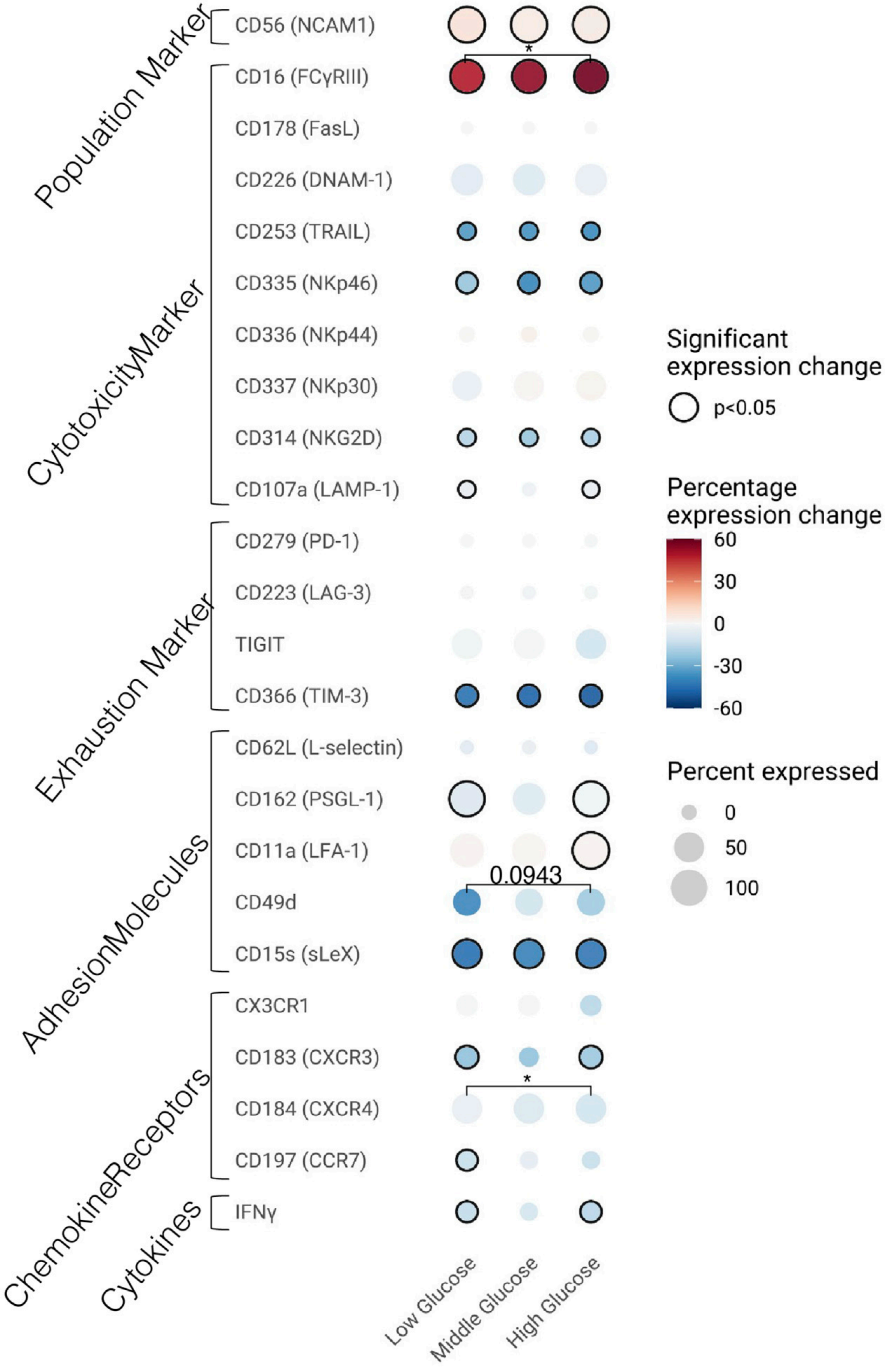
Course of expression levels of surface markers over time during the cultivation process for donor one. Bubble plot based on surface marker expression levels determined with flow cytometry, separated by initial glucose concentrations (low glucose: 5 mM, middle glucose: 8.75 mM, high glucose: 12.5 mM). Percentage expression change: Delta of the percentage of NK cells expressing the marker between the first day of the exponential growth phase and the first day of the stationary phase. Black borders indicate a significant expression change as determined with multiple unpaired t tests. Percent expressed: Bubble size is dependent on percentage of cells expressing the corresponding marker at the day with the highest VCC. Statistics for differences between glucose levels performed by two-way ANOVA with multiple comparisons using the Holm-Šidák method for correction. p < 0.1 is highlighted with the exact value and p < 0.05 is visualized as *.

To observe phenotypical changes of the NK cells during cultivation, NK cells of donor one were analyzed using flow cytometry every other day. Cytotoxicity associated (CD56, CD16, CD178, CD226, CD253, CD335, CD336, CD337, CD314, CD107a) and exhaustion markers (CD279, CD223, CD366, TIGIT), adhesion molecules (CD62L, CD162, CD11a, CD49d, CD15s) as well as chemokine receptors (CX3CR1, CD183, CD184, CD197) were screened, parallel to canonical population markers (CD56, CD3, CD19, CD14) and intracellular IFNγ (Supplementary Table S1). Multiple unpaired t-tests were used to evaluate whether the surface marker expression changes over the cultivation process were significant (Figure 5, black borders). To evaluate the effect of glucose concentration in the cell culture medium on expression levels, a two-way ANOVA was conducted. The activation marker CD253 (TRAIL), CD335 (NKp46) and CD314 (NKG2D) showed a significant decrease in their expression from the beginning of the exponential growth phase until the end of the cultivation (Figure 5) while CD226 (DNAM-1) and CD337 (NKp30) did not change in their respective expression. The population marker CD56 (NCAM1) and the ADCC-facilitating marker CD16 (FCγRIII) showed a significant increase in expression over time. The expression of the exhaustion marker TIM-3 (CD366) decreased greatly over the cultivation phase. CD15s (sLeX) and CD197 (CCR7) were significantly less expressed at the end of the cultivation phase compared to the beginning, regardless of glucose concentration. Overall, these results highlight the impact of the cultivation process and especially the glucose concentration on surface marker and cytokine expression levels.

**FIGURE 6:**
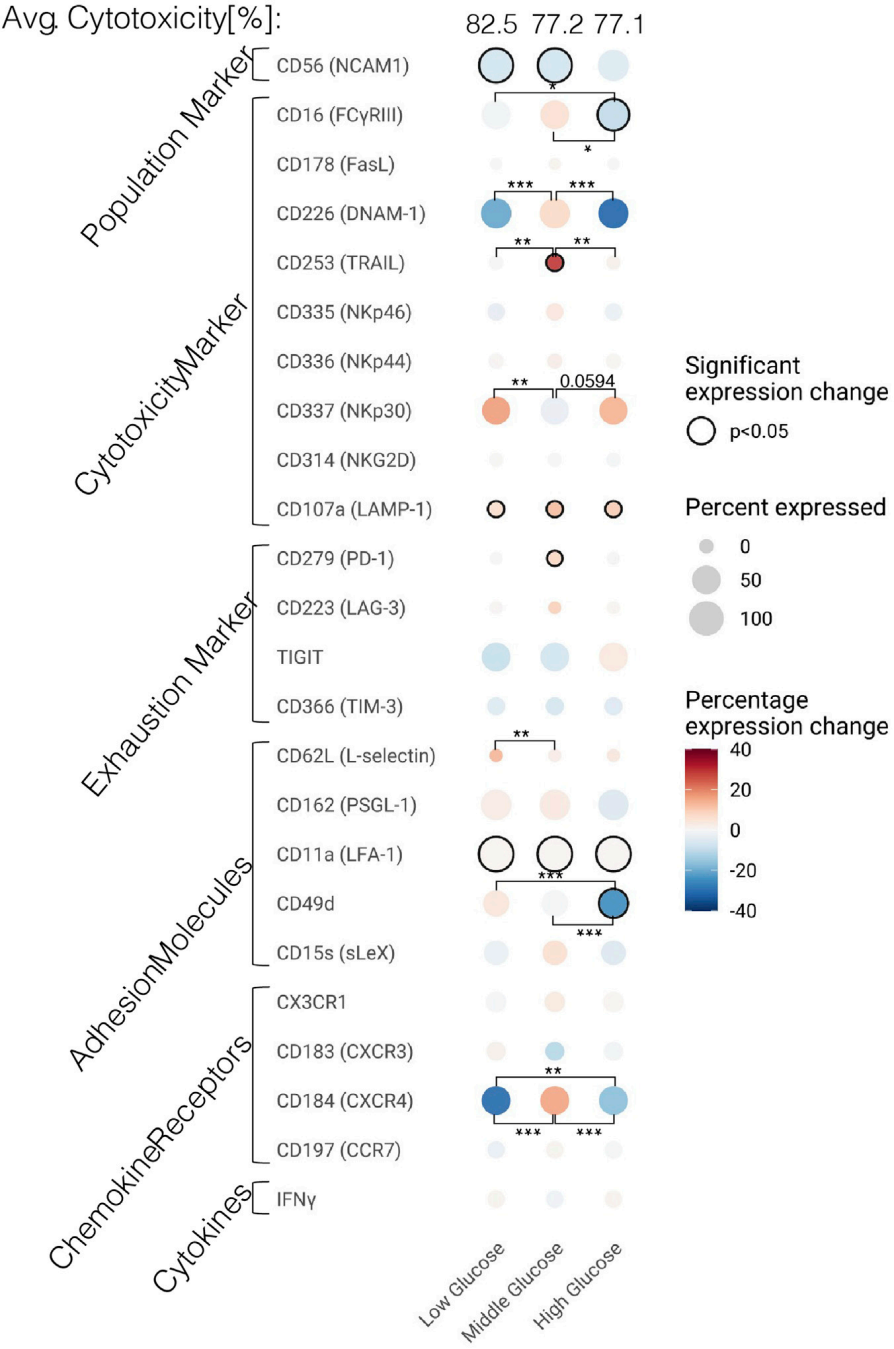
Co-culture effects on expression levels of surface markers. Bubble plot based on surface marker expression levels determined with flow cytometry, separated by initial glucose concentrations (low glucose: 5 mM, middle glucose: 8.75 mM, high glucose: 12.5 mM). Percentage expression change: Delta of the percentage of cells expressing the marker at the day with the highest VCC between cells of the single culture and the co-culture. Black borders indicate a significant expression change as determined with multiple unpaired t tests. Bubble size is dependent on percentage of cells expressing the corresponding marker at the day with the highest VCC. Statistics for differences between glucose levels performed by two-way ANOVA with multiple comparisons using the Holm-Šidák method for correction. p < 0.05 is visualized as *, p < 0.01 as ** and p < 0.001 as ***.

To evaluate the phenotypic effects of co-culturing NK cells with K562 cells, the expression levels between co-culture and single culture NK cells were compared. The comparison was made on the harvesting day with the highest viable cell count (Figure 6). The significance of these differences was determined by multiple unpaired t-tests. A two-way ANOVA was used to determine the impact of glucose levels in the cultivation media on the differences of marker expression between single and co-culture. The co-culture with K562 led to phenotypical changes, especially regarding the activation marker CD107a (LAMP-1), which was significantly higher expressed after co-culture. Cells cultured in higher and lower glucose concentrations behaved similarly with regard to marker expression changes. Both have a decreased expression of CD226 (DNAM-1), CD253 (TRAIL) and CD184 (CXCR4) after 4 hours of co-culture with K562 cells whereas cells cultured in medium glucose concentration show the opposite effects, respectively. The decrease in the expression of CD49d over the co-culture period appears to be connected with the glucose concentration during single culture.

## Discussion

A deeper insight into the relationship between CPPs and CQAs of primary NK cell culture is needed for the understanding and optimization of NK cell expansion. The chosen DoE-based approach allows the identification and differentiation of individual and combined effects of a wide range of metabolic factors. While this methodology has been used in many areas for a long time already and is a standard tool in many microbiological processes, it has not been used according to our knowledge with primary NK cells. We further demonstrate that the expression of multiple cell surface markers is affected throughout the culture process. Our work allows for an unbiased, deep and well-founded insight of *ex vivo* NK cell culture in a longitudinal approach. Moreover, repeated data acquisition enables a phase-dependent culture evaluation in the static culture setup. To improve our analytical workflow, we applied a NODE based approach, which has been described as suitable interpolating sparse timeseries data (Chiu and Du, 2024; Bradley et al., 2024). Because longlasting NK cell culture are inherently sparse and affected by experimental noise, continuous NODE based trajectory reconstruction was used to extract process relevant responses, including maximum VCC, µ and relative change in VCC within the exponential phase.

NK cells from two different donors were used. Donor one was an oncologic patient with breast cancer, while the second was a healthy donor. The two donors were chosen to include donor variability. Notably, the results show similar trends, but more donors are required before a conclusion can be drawn regarding transferability. DoE-1 was planned for a culture period of 12 days at which we expected to reach terminal stationary phase or even the decline phase, based on preliminary experiments. Even though all of the experimental setups with low and middle glucose did indeed reach the stationary phase, a few experimental setups with the high glucose level had not yet reached the stationary phase by the end of the experiment. This leaves the possibility that higher viable cell counts could have been reached if the culture period would have been longer. Consequently, DoE-2 was planned with a 24-day culture duration. This led to a pronounced stationary phase and overall higher cell numbers were achieved. In DoE-1, the highest viable cell count should be interpreted as the maximum that was reached inside the design space and represents the timepoint of transition from the growth phase to the stationary phase. This can lead to some deviations in the comparisons to DoE2 since the biological maximum of VCCmax was not reached for a few runs. Notably, the cytotoxicity of the NK cells remained stable over most of the cultivation period. The change from the exponential growth phase to the stationary growth phase appeared to have no impact on cytotoxicity.

The seeding densities of the cells ranged from 3 to 4.5 x 10^5^. This is due to differences in the recovery of the cells after thawing. The effect was minimized as much as possible but should be considered as a source of variability.

The higher glucose concentrations in DoE-2 did not lead to increased cell numbers. This is most likely associated with the low pH value and/or the high lactate concentration during the culture. It appears that a pH value below 6.5 or a lactate concentration above 25 mM leads to a limitation in cell growth despite the presence of sufficient glucose. Furthermore, high oxygen consumption was observed in DoE-1, where oxygen concentrations dropped to nearly hypoxic conditions, while this effect was less pronounced in DoE-2. The combination of a low pH value, high lactate concentrations and low oxygen concentration, which resulted from long-time stationary culture share similarities with the conditions in the TME. It has been shown that these conditions can influence NK cells in a variety of ways, from decreased cytotoxic activity to blocked IFNγ production (Brand et al., 2016; Husain et al., 2013). Only in DoE-2 an impact of these conditions on cytotoxic activity could be observed due to the longer cultivation period. A controlled culture system (such as in a bioreactor) could avoid an accumulation of lactate and stabilize the pH value and the amount of solubilized oxygen.

Glutamine is absorbed through plasma membrane glutamine transporters and is used in a wide range of biosynthesis processes (Yoo et al., 2020). To investigate this important role, glutamine was varied in the initial concentration in both DoEs. Of note, in all experimental setups, glutamine was consumed but never depleted despite low concentrations in the culture medium. Additionally, glutamine decomposes over time as was observed in controls with pure medium. Overall, glucose consumption completely overshadows the effects of glutamine consumption within DoE-1 with regards to maximal achieved VCC. Interestingly, glutamine and asparagine emerged as important factors when observing the relative change in VCC during the exponential phase. Glutamine was retained for the DoE-2 iteration due to its importance (Terrén et al., 2019; Yoo et al., 2020), even though it has been found to be non-significant. In DoE-2, glutamine was the most important metabolic factor, indicating that at higher concentrations and prolonged cultivation time glutamine emerges as a more important factor than glucose.

Pyruvate plays a crucial role in many metabolic processes and is used in the TCA cycle, which is why it was chosen as one of the varied parameters in DoE-1 (Gray et al., 2014). However, even though pyruvate was consumed, especially in runs with higher pyruvate concentrations, it was not depleted in DoE-1. Even though glucose was completely depleted, it did not lead to further uptake of extracellular pyruvate (Supplementary Figure S2). In DoE-2, pyruvate was completely consumed after longer cultivation time. The exact role of pyruvate in this experimental setup is yet to be determined.

During preliminary experiments using the NK92 cell line, serine consumption during expansion was observed (data not shown). To further investigate this and to determine the impact of serine concentrations in the culture medium, the concentration ranges of serine were varied in DoE-1. Serine was indeed consumed during cell culture, albeit not completely depleted. Our results indicate an impact of serine concentration on the accumulation of formic acid, with higher serine levels leading to higher formic acid accumulation. Evidence of an impact of serine consumption and formic acid accumulation on any of the responses could not be found.

Asparagine was varied as well during DoE-1 but was not consumed. None of the other amino acids that were measured appear to cause any limitations. Similarly, asparagine was consumed by NK92 cells during preliminary experiments and therefore chosen as a variable parameter (data not shown). The primary NK cells did not consume asparagine nor showed the different initial concentrations any discernible effect. Asparagine occurred as an influencing factor within DoE-1 when observing the relative change within the exponential phase.

To get a better understanding of phenotypic changes during cell culture flow cytometric analyses were performed every other day with a wide panel covering many canonical activation markers, exhaustion markers, and more. Many markers showed no difference in expression changes regarding the initial glucose concentration. However, CD16 (FCγRIII) increased in every experimental run over time and significantly more in runs with higher glucose compared to runs with lower initial glucose. This could indicate a maturation of NK cells to the CD56^dim^ CD16^+^ phenotype, which may be increased by higher glucose concentration (Sivori et al., 2021). Unfortunately, the clone of the CD56 targeting antibody does not allow for a differentiation between CD56^bright^ and CD56^dim^, which could have helped to investigate this hypothesis. While two cytotoxic markers, TRAIL and NKp46, decrease in their expression over time, other cytotoxic markers like DNAM-1 and NKp30 remain highly expressed throughout the culture period. Overall, the phenotypic changes that were discovered appear to have no impact on the cytotoxic activity of the NK cells against K562 cells.

During the 4-h co-culture period with K562 cells, strong phenotypic changes occurred. Cells that were cultivated with middle concentrations of glucose partially show completely different expression changes compared to cells cultured in higher or lower glucose concentrations. This affects cytotoxicity markers, adhesion molecules, and chemokine receptors. Notably, overall cytotoxicity was not significantly affected and diminished. More functional data is required to be able to draw conclusions regarding the impact of these marker changes.

In these experiments, two consecutive DoEs were performed, where PLS models were used to identify metabolic factors affecting VCC. The DoE-based approach enabled insight into the relationship between relevant CPPs and CQAs, which helped to develop a data-driven representation of primary NK-cell behavior. However, the presented analysis represents the initial steps in model formulation, as the ultimate goal of the generated data is to be used to generate a mechanistic model for NK-cell growth. Finally, a digital twin-assisted process development framework could be established to enable knowledge-based and donor specific manufacturing processes with the demonstration of increased immune activity after application of digital twin suggested adaptations of the culture conditions. Even though these tools are partly established for production of biopharmaceuticals and encouraged by the FDA, they have hardly been applied yet to manufacturing cell therapeutics. An approach in this respect was suggested by Stacey et al. (Stacey et al., 2018).

In total, our DoE-based workflow allows for the identification of CPP’s and their impact on CQAs. This structured experimental approach also facilitates the formulation of a data-driven model for a better representation of the primary NK cells’ behavior which can help to deliver further insights for process understanding. Our results also point out that certain cell surface markers may dramatically change over the course of the culture, which should be considered in future clinical studies. Future work will focus on a more intensive investigation of the identified cause-effect relationships and the mechanistic formulation of NK cell growth.

## Data Availability

The datasets presented in this study can be found in online repositories. The names of the repository/repositories and accession number(s) can be found below: The data that support the findings of this study are openly available in Zenodo at http://doi.org/10.5281/zenodo.18832899.
